# FLU-v, a Broad-Spectrum Influenza Vaccine, Induces Cross-Reactive Cellular Immune Responses in Humans Measured by Dual IFN-γ and Granzyme B ELISpot Assay

**DOI:** 10.3390/vaccines10091528

**Published:** 2022-09-14

**Authors:** Fredrik Oftung, Lisbeth M. Næss, Ida Laake, Gregory Stoloff, Olga Pleguezuelos

**Affiliations:** 1 Department of Method Development and Analytics, Division of Infectious Disease Control, Norwegian Institute of Public Health, P.O. Box 222, N-0213 Oslo, Norway; 2 Department of Infection Control and Vaccines, Division of Infectious Disease Control, Norwegian Institute of Public Health, P.O. Box 222, N-0213 Oslo, Norway; 3 SEEK, London Bioscience Innovation Centre, 2 Royal College St, London NW1 0NH, UK; 4 ConserV Bioscience, 77 Heyford Park, Bicester OX25 5HD, UK

**Keywords:** broad spectrum influenza vaccine, FLU-v, clinical trial, dual ELISpot, cellular immune responses, cross-reactivity

## Abstract

Previous reports demonstrated that FLU-v, a peptide-based broad-spectrum influenza vaccine candidate, induced antibody and cellular immune responses in humans. Here, we evaluate cellular effector functions and cross-reactivity. PBMC sampled pre- (day 0) and post-vaccination (days 42 and 180) from vaccine (*n* = 58) and placebo (*n* = 27) recipients were tested *in vitro* for responses to FLU-v and inactivated influenza strains (A/H3N2, A/H1N1, A/H5N1, A/H7N9, B/Yamagata) using IFN-*γ* and granzyme B ELISpot. FLU-v induced a significant increase in the number of IFN-γ- and granzyme-B-secreting cells responding to the vaccine antigens from pre-vaccination (medians: 5 SFU/10^6^ cells for both markers) to day 42 (125 and 40 SFU/10^6^ cells, *p* < 0.0001 for both) and day 180 (75 and 20 SFU/10^6^ cells, *p* < 0.0001 and *p* = 0.0047). The fold increase from pre-vaccination to day 42 for IFN-γ-, granzyme-B-, and double-positive-secreting cells responding to FLU-v was significantly elevated compared to placebo (medians: 16.3-fold vs. 1.0-fold, *p* < 0.0001; 3.5-fold vs. 1.0-fold, *p* < 0.0001; 3.0-fold vs. 1.0-fold, *p* = 0.0012, respectively). Stimulation of PBMC with inactivated influenza strains showed significantly higher fold increases from pre-vaccination to day 42 in the vaccine group compared to placebo for IFN-γ-secreting cells reacting to H1N1 (medians: 2.3-fold vs. 0.8-fold, *p* = 0.0083), H3N2 (1.7-fold vs. 0.8-fold, *p* = 0.0178), and H5N1 (1.7-fold vs. 1.0-fold, *p* = 0.0441); for granzyme B secreting cells reacting to H1N1 (3.5-fold vs. 1.0-fold, *p* = 0.0075); and for double positive cells reacting to H1N1 (2.9-fold vs. 1.0-fold, *p* = 0.0219), H3N2 (1.7-fold vs. 0.9-fold, *p* = 0.0136), and the B strain (2.0-fold vs. 0.8-fold, *p* = 0.0227). The correlation observed between number of cells secreting IFN-γ or granzyme B in response to FLU-v and to the influenza strains supported vaccine-induced cross-reactivity. In conclusion, adjuvanted FLU-v vaccination induced cross-reactive cellular responses with cytotoxic capacity, further supporting the development of FLU-v as a broad-spectrum influenza vaccine.

## 1. Introduction 

Seasonal influenza virus continues to cause high morbidity and mortality every year, and sporadic epidemics and pandemics cause additional burdens to health care systems worldwide. For decades, seasonal flu vaccines have included hemagglutinin (HA) isolated from the strains predicted to be in circulation in the coming season as the principal vaccine antigen. Due to the high mutation rate in the HA gene of influenza A and B viruses, updates to the vaccine are required to match this antigenic drift every year [1]. In addition, even more abrupt antigenic shifts, often involving re-assortment of the major surface antigens, can create new viruses with pandemic potential due to a lack of strain-specific protective immunity in the population [1]. Influenza vaccines that provide broad protection against diverse influenza strains are, therefore, highly needed to provide seasonal protection and pandemic preparedness against emergent influenza strains [1]. Several different approaches to develop broad spectrum influenza vaccines by targeting conserved regions of various influenza antigens to induce protective antibody and/or cell-mediated immune responses are being evaluated in clinical trials [2,3,4,5]. In this context, virus-like particles, protein or peptide-based subunit vaccines, virus-vector-based vaccines, and DNA/RNA constructs are among the most explored vaccine platform technologies for the efficient induction of both antibody and cellular immunity [2]. 

FLU-v is an adjuvanted peptide vaccine consisting of four short peptides originating from conserved regions of the M1, M2, NP-A, and NP-B proteins of the influenza virus that are manufactured using F-moc chemistry. Previous reports demonstrated that FLU-v induced cellular and humoral immune responses in both preclinical [4] and clinical studies [5,6,7], and reduced mild-to-moderate influenza disease in a human challenge study [8,9]. FLU-v immune responses were evaluated in a phase IIb clinical trial within the UNISEC Consortium framework [10]. The evaluation of cellular immune responses showed that a single dose of adjuvanted FLU-v was more effective than two doses of non-adjuvanted FLU-v in stimulating T cells positive for IFN-γ, TNFα, or IL-2 measured by multi-parametric flow cytometry [5]. Moreover, adjuvanted vaccination also induced strong FLU-v-specific IgG responses that could play a role in antibody-dependent cellular cytotoxicity (ADCC) or complement activation against influenza-infected cells [5]. 

Utilizing leftover cryopreserved PBMC samples from the adjuvanted FLU-v group and adjuvanted placebo from the above-mentioned clinical phase IIb trial [5], we aimed to explore vaccine-induced effector functions and cross-reactive cellular immunity by means of measuring IFN-γ- and granzyme-B-producing cells by dual ELISpot assay in response to a panel of diverse influenza strains. 

## 2. Materials and Methods

### 2.1. Vaccination

FLU-v is a 200 nM equimolar mixture of four lyophilized synthetic peptides (Table 1) originated from conserved regions of the M1, M2, NP-A, and NP-B influenza virus proteins [5]. The adjuvanted formulation is prepared by reconstituting lyophilized FLU-v in 0.25 mL of Montanide ISA-51 (Seppic) and 0.25 mL of water for injection, and mixing to create a water-in-oil emulsion. The adjuvanted placebo formulation consisted of 0.5 mL of water-in-oil emulsion without FLU-v. Montanide ISA-51 is made of a mineral oil and a surfactant designed to increase antigen specific immune responses [11]. Healthy adult volunteers in the vaccine (*n* = 58) or placebo (*n* = 27) groups received a subcutaneous injection of adjuvanted FLU-v or adjuvanted placebo on day 0, followed by a saline injection on day 21, as determined by the randomization code. Study approval was obtained after ethical review by the Dutch Central Committee on Research Involving Human Subjects (reference NL55061.000.15), followed by approval from the competent authority (the Dutch Ministry of Health, Welfare and Sport). Informed consent was obtained from each participant before proceeding with the trial interventions. The study was registered in EudraCT: 2015-001932-38 [6,8].

### 2.2. Dual ELISpot Assay 

PBMC was isolated from blood samples taken from all volunteers on day 0 (pre-vaccination), and days 42 and 180 post-vaccination. Cryo-preserved PBMC were thawed and washed in RPMI 1640 (15% FCS), and left to recover overnight in 5 mL RPMI 1640 (15% FCS and 100 U/mL penicillin/streptomycin) in a 37 °C humidified 5% CO_2_ incubator. Viable cells were counted in a MUSE automated cell counter (Millipore) using MUSE count and viability kit (Millipore). Viable cells were adjusted to 1 million cells/mL in RPMI 1640 (15% FCS) to reach a final concentration of 100,000 cells/100 μL/well. All samples representing different time points from the same participant were tested on the same plate. 

FLU-v vaccine antigens were added to the cells as a mixture of the four synthetic peptides to reach a final concentration of 4 μM/well (total peptide concentration). Phytohemagglutinin (PHA-P) was added to the assay as positive control at a final concentration of 2 μg/mL, and complete cell culture medium (RPMI 1640 with 15% FCS) was used as negative control. Based on previous in-house titration experiments, 100 hemagglutinin units (HAU)/mL of each inactivated influenza virus strain was used as the final concentration. The inactivated strains used included A/California/7/2009 (H1N1), A/Shanghai/24/1990 (H3N2), B/Brisbane/9/2014 (Yamagata lineage), A/Vietnam/1194/2004 (NIBRG-14) (H5N1), and A/Anhui/1/2013 (NIBRG-268) (H7N9), all obtained from the National Institute for Biological Standards and Controls (NIBSC, United Kingdom). All antigens and controls were assayed in duplicates. Plates were incubated for 24 h in a 37 °C humidified 5% CO_2_ incubator. 

A human IFN-γ and granzyme B double color enzymatic ELISpot kit (Cellular Technology Limited, Bonn, Germany) was used to enumerate the cells secreting IFN-γ and granzyme B after *in vitro* antigen stimulation. This assay allows for the simultaneous detection of two important markers related to protective immune responses against influenza. Dual detection of these parameters was performed by using a FITC-conjugated detection antibody for IFN-γ and a biotin-conjugated detection antibody for granzyme B, followed up with FITC-HRP and Strep-AP incubation for the visualization of red (INF-γ) and blue (granzyme B) spots, respectively. Double positive cells expressing both immune markers were detected as pink spots. The assay was performed following the manufacturer’s instructions. 

Spot forming units (SFU) were counted using the Immuno-Spot Series 6 Ultra-V plate analyzer (Cellular Technology Ltd.). This instrument was ISO certified (ISO 9001:2008), and counting options and procedures followed GCLP guidelines. The mean SFU for each duplicate sample was calculated and normalized by subtracting mean SFU counts for the negative control. Normalized SFU counts with values ≤ 0 were assigned a value of 0.5, and counts were finally multiplied by 10 to present the data as SFU/million cells. Samples not meeting the acceptance criteria for positive control (PHA stimulation > 20 SFU/10^5^ cells) or negative control (medium only < 10 SFU/10^5^ cells) were excluded from the statistical analysis. The discrepancies between the number of participants recruited to the study and the number of data points finally included in statistical analysis were due to missing donor visits and samples, an insufficient number of viable cells in the sample to test all antigens, and an inability to meet the acceptance criteria for positive and negative controls in the assay.

### 2.3. Statistical Analyses 

The Wilcoxon signed rank sum test was used to compare the number of cells over time within each group, whereas the Mann–Whitney U test was used to compare the fold-increase between groups at the same time point. Non-parametric tests were chosen because the D’Agostino and Pearson Omnibus test and visual inspection of the histograms and QQ-plots demonstrated that the data were not normally distributed. Vaccine responders were defined as participants with an SFU count of at least 40 SFU/million cells on days 42 or 180 post-vaccination, and showing a minimum of a two-fold increase in response from pre-vaccination to post-vaccination. *p*-values for comparison of percentage of responders between groups were calculated with Fisher’s mid-P test. Spearman’s rank correlation coefficients between the number of IFN-γ- or granzyme-B-secreting cells in response to the vaccine antigens and the different inactivated influenza strains were calculated. Statistical analyses were performed using Stata SE 16.0 (StataCorp LLC, College Station, TX, USA) and GraphPad 8.1.2 (Dotmatics, Boston, MA, USA) software.

## 3. Results

Two different data analyses were used to facilitate the interpretation of the responses. Firstly, comparisons were made between the median number of positive cells at the different time points within the groups, allowing visualization of the pre-vaccination background levels. Secondly, the fold increase from pre-vaccination to post-vaccination was calculated and compared to placebo to show the vaccine effect more accurately. 

### 3.1. ELISpot Responses to FLU-v

In the FLU-v vaccinated group, the number of IFN-γ-producing cells detected after vaccine antigen stimulation was significantly higher on day 42 (median: 125 SFU/million cells, *p* < 0.0001) and day 180 (median: 75 SFU/million cells, *p* < 0.0001) after vaccination compared to pre-vaccination (median: 5 SFU/million cells) (Table 2). Significantly higher levels of FLU-v-specific granzyme-B-producing cells were also observed on day 42 (median: 40 SFU/million cells, *p* < 0.0001) and day 180 (median: 20 SFU/million cells, *p* < 0.0047) compared to pre-vaccination (median: 5 SFU/million cells) (Table 2). Finally, the number of vaccine-antigen-specific cells secreting both IFN-γ and granzyme B was significantly higher on day 42 (median 20 SFU/million cells, *p* < 0.0001) and day 180 (5 SFU/million cells, *p* = 0.0059) compared to pre-vaccination. However, the median number of double positive cells was the same on day 180 as pre-vaccination, since the minimum possible number of cells was observed on both time points for about half of the participants. No significant differences between pre- and post-vaccination were observed in the adjuvanted placebo group for any of the immune markers tested (Table 2). 

Significant differences in the fold-increase between the FLU-v vaccinated group and the placebo group were seen for IFN-γ-producing cells from day 0 to day 42 (medians: 16.3-fold vs. 1.0-fold, *p* < 0.0001) and to day 180 (medians: 9.5-fold vs. 1.0-fold, *p* < 0.0001) (Table 3). The corresponding fold increases from pre- to post-vaccination for granzyme-B-secreting cells in the vaccine group were lower than those observed for the IFN-γ-secreting cells, but still significantly higher than in the placebo group both on day 42 (medians: 3.5-fold vs. 1.0-fold, *p* < 0.0001) and on day 180 (medians: 2.0-fold vs. 1.0-fold, *p* = 0.0461) (Table 3). The fold increase for double positive cells in the FLU-v group was only significantly different from placebo on day 42 (medians: 3.0-fold vs. 1.0-fold, *p* = 0.0012) (Table 3). 

In the FLU-v vaccinated group, the percentage of responders to the vaccine antigens was 75% (95% CI 61.2–85.1) on day 42 and 62.5% (95% CI 47–75.8) on day 180, based on IFN-γ-secreting cells; 54.3% (95% CI 40.2–67.8) on day 42 and 29.3% (95% CI 17.6–44.5) on day 180 for granzyme-B-secreting cells; and 32% (95% CI 17.2–51.6) on day 42 and 19% (95% CI 7.7–40.0) on day 180 for double positive cells. Based on the responder definition used, no responders were detected in the placebo group for any of the markers used on day 42, whereas 6% were responders for IFN-γ and granzyme B on day 180.

### 3.2. ELISpot Responses to a Panel of Influenza Strains

To evaluate the ability of the FLU-v vaccine to induce cross-reactive cellular immune responses, we also included a panel of relevant seasonal and pandemic inactivated influenza A and B strains when testing PBMC in ELISpot assays. Unlike the low baseline stimulation levels seen for the FLU-v antigens, higher levels of IFN-γ- and granzyme-B-producing cells responding to the influenza strains were already detected pre-vaccination in both groups (Table 2). Despite this, vaccination with adjuvanted FLU-v still induced a significant increase in the number of IFN-γ-secreting cells (SFU/million) from day 0 to day 42 in response to all five virus strains tested (medians for H1N1: 128 vs. 310, *p* = 0.0001; H3N2: 268 vs. 555, *p* = 0.0001; H5N1: 460 vs. 668, *p* = 0.0001; H7N9: 130 vs. 268, *p* = 0.0026; and the B strain: 130 vs. 243, *p* = 0.0085) (Table 2). From day 0 to day 180, significant differences in the number of IFN-γ-producing cells (SFU/million) were only observed for the H1N1 (medians: 128 vs. 240, *p* < 0.0163) and H5N1 (medians: 460 vs. 585, *p* = 0.0381) strains. In addition, significant increases in the number of granzyme-B-secreting cells (SFU/million) from pre-vaccination to day 42 were also seen upon *in vitro* stimulation with the H1N1 (medians: 50 vs. 240, *p* < 0.0001), H3N2 (medians: 193 vs. 485, *p* = 0.0002), and H5N1 (medians: 305 vs. 650, *p* = 0.0041) strains. Although increases were also seen from day 0 to day 180, these were not statistically significant (Table 2). Finally, an analysis of the double positive cell population, expressing both IFN-γ and granzyme B, showed significantly higher cell numbers (SFU/million) on day 42 than on day 0 for H1N1 (medians: 25 vs. 125, *p* < 0.0011), H3N2 (medians: 135 vs. 220, *p* = 0.0064), H5N1 (medians: 245 vs. 305, *p* = 0.0220), and B influenza (medians: 10 vs. 30. *p* = 0.0046), as well as H1N1 (medians: 25 vs. 50, *p* = 0.0025) and H3N2 (medians: 135 vs. 205, *p* = 0.0449) on day 180 after vaccination. (Table 2). No differences in the placebo group were observed between pre- and post-vaccination in the number of cells secreting IFN-γ, granzyme B, or both factors with any of the influenza strains tested (Table 2).

The overall IFN-γ fold increase levels from pre- to post-vaccination in the vaccinated group after stimulation with the influenza strains were lower than the response seen after stimulation with the vaccine antigens, but still significantly higher than in the placebo group on day 42 for H1N1 (medians: 2.3-fold vs. 0.8-fold, *p* = 0.0083), H3N2 (medians: 1.7-fold vs. 0.8-fold, *p* = 0.0178), and H5N1 (medians: 1.7 vs. 1.0, *p* = 0.0441) (Table 3). Adjuvanted FLU-v vaccination also induced a significantly higher fold increase for granzyme-B-producing cells in response to H1N1 (medians: 3.5-fold vs. 1.0-fold, *p* = 0.0075) on day 42 compared to placebo (Table 3). Moreover, analysis of the double positive cell population showed a significant difference in fold increase between the vaccine and placebo group for H1N1 (medians: 2.9-fold vs. 1.0-fold, *p* = 0.0219), H3N2 (medians: 1.7-fold vs. 0.9-fold, *p* = 0.0136), and the B strain (medians: 2.0-fold vs. 0.8-fold, *p* = 0.0227) on day 42 after vaccination (Table 3). No significant differences in fold increase from baseline to day 180 were observed between the vaccine and placebo groups for any of the strains tested.

### 3.3. Correlation Analysis

**Figure 1:**
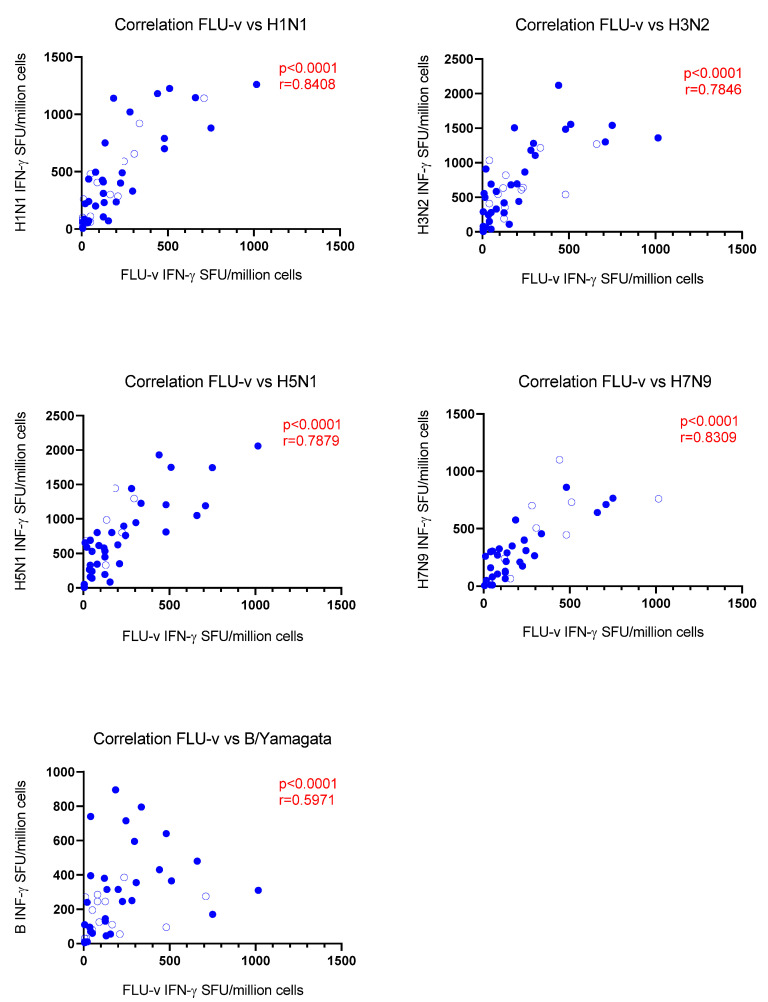
Correlation analysis between the number of IFN-γ-secreting cells (SFU/million cells) in response to the equimolar mix of the four synthetic peptides included in the FLU-v vaccine and in response to whole inactivated influenza strains (A/California/7/2009 (H1N1), A/Shanghai/24/1990 (H3N2), A/Vietnam/1194/2004 (NIBRG-14) (H5N1), A/Anhui/1/2013 (NIBRG-268) (H7N9), and B/Brisbane/9/2014 (Yamagata lineage)) on day 42 in the FLU-v vaccinated group. Correlation coefficients (r) and *p*-values according to the Spearman analysis are indicated.

**Figure 2:**
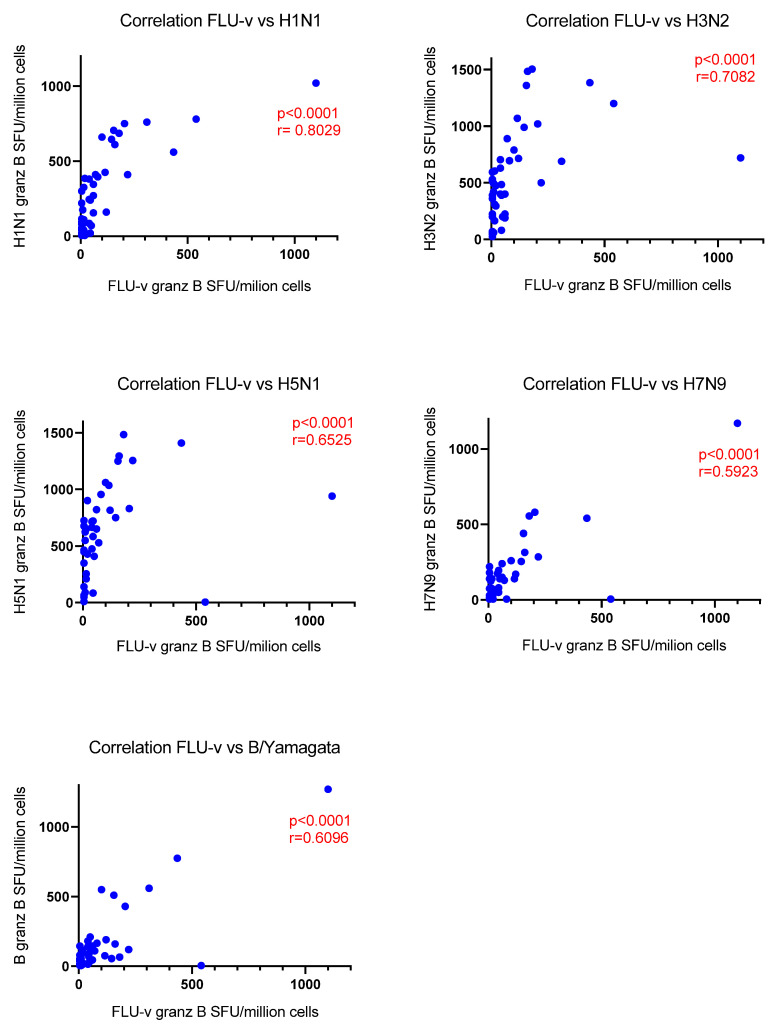
Correlation analysis between the number of granzyme-B-secreting cells (SFU/million cells) in response to the equimolar mix of the four synthetic peptides included in the FLU-v vaccine and in response to whole inactivated influenza strains (A/California/7/2009 (H1N1), A/Shanghai/24/1990 (H3N2), A/Vietnam/1194/2004 (NIBRG-14) (H5N1), A/Anhui/1/2013 (NIBRG-268) (H7N9), and B/Brisbane/9/2014 (Yamagata lineage)) on day 42 in the FLU-v vaccinated group. Correlation coefficients (r) and *p*-values according to the Spearman analysis are indicated.

Correlation analyses were performed between the number of cells secreting the different markers detected on day 42 after stimulation with FLU-v antigens and stimulation with the individual strains in vaccinated individuals to determine whether the cellular responses to the FLU-v antigens were associated with responses to the influenza strains. Strong correlations were observed for the number of cells secreting INF-γ in response to FLU-v and in response to the A influenza strains, but to a lesser extent to the B strain (H1N1: r = 0.84, H3N2: r = 0.78, H5N1: r = 0.79, H7N9: r = 0.83, B: r = 0.60, *p* < 0.0001 for all correlations) (Figure 1). We also found similar correlations between the number of granzyme-B-producing cells after stimulation with FLU-v and the influenza strains (H1N1: r = 0.80, H3N2: r = 0.71, H5N1: r = 0.65, H7N9: r = 0.59, B: r = 0.61, *p* < 0.0001 for all correlations) (Figure 2), but not for double positive cells.

## 4. Discussion

Many efforts have been made over the years to make influenza vaccines more effective. Numerous attempts have concentrated on improving the duration and titer of the antibody response [12]. Although representing a valid short-term improvement, this is not a sustainable long-term solution to the problem of antigenic drift and shift, and achieving broader protection continues to be a major goal [2,10]. The peptide-based FLU-v vaccine candidate aims to address this problem by combining short, conserved regions of the influenza proteins, M1, M2, and NP [4]. Previous studies have shown that FLU-v induces both antibodies and cellular immune responses in pre-clinical [4] and clinical settings [5,6,7,9], as well as protection against mild-to-moderate influenza disease in a human challenge study [9]. FLU-v-specific antibodies are not expected to be neutralizing, as they do not target epitopes on the viral surface, but they could bind to infected cells, triggering a cytotoxic response by means of activating complement or ADCC responses. The cellular immune responses triggered by FLU-v vaccination are biased towards Th1, as previously measured by multi-parametric flow cytometry and cytokine ELISA [5], and the IgG antibodies generated include both IgG1 and IgG3 subclass components (unpublished data), which are relevant for ADCC responses. To further evaluate cellular effector functions and vaccine-induced cross-reactivity, PBMC samples from the UNISEC study [5,6] were assessed for *in vitro* responses to FLU-v antigens and a panel of inactivated heterosubtypic influenza strains by means of using dual ELISpot to enumerate IFN-γ- and granzyme-B-producing cells as markers for cytotoxic cellular responses.

As demonstrated in this study, adjuvanted FLU-v vaccination induced a significant increase in the number of vaccine-specific IFN-γ- and granzyme-B-secreting cells in response to the FLU-v antigens. Interestingly, the responses to FLU-v antigens were low pre-vaccination and in the placebo group, indicating that natural exposure to influenza does not generate significant cellular responses to the viral protein regions covered by FLU-v, but they are induced through vaccination. A wide range of protein epitopes compete for binding to MHC class I and II molecules during an infection, leading to CD8+ and CD4+ T cell activation, respectively. Immunodominant epitopes are more successful in being presented to the immune system than subdominant epitopes due to the intrinsic characteristics of the epitope sequences, genetic factors such as MHC alleles, and how the antigens are processed [13,14]. However, epitope binding to MHC molecules does not always translate into activation of cells, leading to protective or cross-reactive responses. The FLU-v vaccine trains the immune system to respond to conserved epitopes that would normally not be presented due to competition with more immunodominant epitopes, which explains why a response is observed only after vaccination. Others have shown that vaccination with subdominant epitopes is a viable approach for inducing protection against respiratory viral infections [15,16]. Vaccination targeting non-dominant epitopes alone facilitated a broader and more potent response against murine lymphocytic choriomeningitis virus as compared to adding immunodominant epitopes [17].

As shown in this work, *in vitro* testing of PBMC for responses to heterosubtypic influenza strains confirmed that FLU-v vaccination not only generates an immune response able to recognize naturally processed and presented influenza antigens, but also demonstrated that these responses are cross-reactive for diverse influenza A and B strains. A critical step in the development of broad-spectrum influenza vaccines is to demonstrate the induction of cross-reactive cellular responses contributing to protection from a variety of seasonal and pandemic influenza strains. However, demonstrating cross-protection in a clinical setting is challenging due to the fact that only a limited number of influenza subtypes circulate in the human population within a given season, and the emergence of those strains that pose a pandemic threat is difficult to predict. It is possible to perform controlled human challenge studies, but only a handful of relevant strains have been approved for infection of volunteers. This situation calls for alternative approaches to demonstrate the broadness of cellular immunity. Employing *in vitro* immune assays to measure well established surrogate markers of protection may allow for the evaluation of predicted efficacy against any influenza strains of interest, including those with pandemic potential isolated from animals. As a step in this direction, we have, in this study, demonstrated that cellular immune responses induced by FLU-v vaccination cross-recognized all influenza strains tested (H1N1, H3N2, H5N1, H7N9, and B influenza), as measured by an increase in IFN-γ- and granzyme-B-secreting cells detected *in vitro*. Additional support for vaccine-induced cross-reactive cellular immune responses was obtained by demonstrating a direct correlation between the responses to FLU-v and the responses to the individual viral strains. Cross-recognition of the strains tested here is of importance because H1N1 and H3N2 have been the main circulating seasonal strains for many years [18], whereas H5N1 and H7N9 are strains of high concern due to their pandemic potential [19,20].

In contrast to the low background levels of responses to FLU-v antigens prior to vaccination, IFN-γ and granzyme B responses to the influenza strains were elevated before vaccination, thereby reducing the fold increase from pre- to post-vaccination. Although the innate responses to viral components may have contributed to higher pre-vaccination levels, it is also likely that adaptive cross-priming with naturally circulating seasonal influenza (H1N1 and H3N2) has induced pre-immunity to other strains not circulating among the study participants [21,22]. Naturally occurring cells cross-reacting with influenza strains not normally circulating in the tested population has also been observed by others [22,23,24,25]. The presence of pre-immunity at baseline may have reduced the ability to detect the true potential of FLU-v vaccination to induce cross-reactive cellular responses with the assay conditions used here. It is also likely that the use of live virus stimulation or transfection of target cells with inactivated virus would represent a more efficient methodological approach for detecting CD8+ T cell responses, which are the main cell type with cytotoxic potential. This was not possible due to biosafety restrictions.

The mode of action for the current seasonal influenza vaccines is the generation of neutralizing antibodies against the major surface glycoprotein hemagglutinin (HA), commonly quantified by the hemagglutinin inhibition (HI) assay and used as the traditional correlate of protection [3,26]. However, evaluation of universal influenza vaccines that work by activating T-cell responses requires alternative correlates of protection that should be standardized to be suitable for use in large multicenter clinical trials [3,26,27]. ELISpot is a functional, quantitative, and sensitive assay for the detection of cytokines and other immune markers at the single cell level, providing a suitable tool for assessing the immunogenicity and biomarkers of vaccine efficacy in clinical trials [28]. The dual ELISpot assay used here allows for the rapid measurement of IFN-γ-producing cells as a marker of Th1 responses, and granzyme-B-producing cells as a marker of cytotoxicity. Importantly, both of these immune parameters are associated with cell-mediated protection against influenza disease [3,29]. It has been shown that the presence of both CD8+ and CD4+ T cells producing IFN-γ correlated with a low total symptom score after infection [30,31], and viral clearance and reduced shedding in the absence of specific antibodies [32]. Granzyme B contributes to protection by signaling the elimination of virus-infected host cells [33,34] using the apoptotic pathway, resulting in DNA fragmentation and a rapid loss of membrane integrity [35]. Although ELISpot does not discriminate between the different cell phenotypes, previous work indicated that CD8+ T cells with cytotoxic potential were induced by FLU-v vaccination, as detected by transfection of FLU-v into target cells exposed to splenocytes from vaccinated mice [4]. Moreover, depletion of CD8+ T cells resulted in reduced IFN-γ secretion from PBMC in FLU-v vaccinated human volunteers [8]. Low CD8+ T cell stimulation *in vitro,* as previously reported for this clinical study [5], may most likely be due to the assay conditions not being optimal for efficient presentation of FLU-v antigens by MHC class I molecules rather than a lack of response. The efficacy of FLU-v was tested in an H1N1 challenge study in human volunteers, and a single dose of adjuvanted FLU-v was more effective in reducing mild-to-moderate disease than two doses of adjuvanted FLU-v [9]; therefore, increasing the number of doses seems unlikely to provide additional benefits, at least as measured with these parameters in a short-term perspective.

The reported IFN-γ response triggered by the FLU-v antigens in this study is consistent with previous results obtained with other immunological assays, such as IFN-γ ELISA and multi-parametric flow-cytometry [5]. Detection of granzyme-B-secreting cells in vaccinated subjects provides additional evidence for the induction of cellular responses with cytotoxic capacity after FLU-v vaccination. In addition, detection of double positive cells, although at a low frequency, indicates that FLU-v activates a cell type that exhibits multifunctional properties and may play an important role in protection against influenza infection [36].

In summary, this study reports that adjuvanted FLU-v vaccination can induce cross-reactive cellular responses with potential cytotoxic capacity, as detected by dual IFN-γ and granzyme B ELISpot assays. Moreover, the results suggest that further optimization and usage of such *in vitro* assays may serve as a standardized approach to evaluate cross-reactive cell-mediated immune responses in clinical testing of universal vaccines, with options for assessing even potential pandemic strains not yet in human circulation. In conclusion, the data show that adjuvanted FLU-v is a promising broad-spectrum influenza vaccine candidate that warrants further testing for protective efficacy against disease in clinical phase III trials.

## Figures and Tables

**Table 1:** Amino acid sequences of the peptides included in the FLU-v vaccine and the protein antigens they originate from.

Peptide Name	Protein Origin	Amino Acid Sequence
FLU-5 acetate	M1 protein	DLEALMEWLKTRPILSPLTKGILGFVFTLTVP
FLU-7 acetate	NP protein from A strains	DLIFLARSALILRGSVAHKS
FLU-8N acetate	NP protein from B strains	PGIADIEDLTLLARSMVVVR
FLU-10 acetate	M2 protein	IIGILHLILWILDRLFFKCIYRLF

**Table 2:** Median number of IFN-γ-secreting, granzyme-B-secreting, and double positive cells (SFU/million cells) in response to FLU-v and whole inactivated influenza strains (A/California/7/2009 (H1N1), A/Shanghai/24/1990 (H3N2), A/Vietnam/1194/2004 (NIBRG-14) (H5N1), A/Anhui/1/2013 (NIBRG-268) (H7N9), and B/Brisbane/9/2014 (Yamagata lineage)) in the group vaccinated with adjuvanted FLU-v and the placebo group on days 0, 42, and 180. *n* = number of samples to be included in statistical analysis. CI = 95% confidence interval. Wilcoxon signed rank sum test was used to analyze differences between day 0 and 42 and between day 0 and 180 in each group. Significant differences are indicated with *p*-values in bold.

Antigen	Treatment Group	Median SFU (n) (CI) Day 0 IFN-γ Granzyme B Double Positive	Median SFU (n) (CI) Day 42 IFN-γ Granzyme B Double Positive	Median SFU (n) (CI) Day 180 IFN-γ Granzyme B Double Positive	*p*-Value (Wilcoxon) Day 0–42	*p*-Value (Wilcoxon) Day 0–180
**FLU-v**	**Adjuvanted FLU-v**	**5** (49) (5–5)	**125** (48) (50–200)	**75** (40) (40–130)	**<0.0001**	**<0.0001**
		**5** (48) (5–5)	**40** (46) (10–60)	**20** (41) (5–40)	**<0.0001**	**0.0047**
		**5** (28) (5–5)	**20** (25) (10–40)	**5** (21) (5–20)	**<0.0001**	**0.0059**
	**Adjuvanted** **Placebo**	**5** (22) (5–10)	**5** (21) (5–10)	**5** (17) (5–15)	0.83	0.14
		**5** (20) (5–10)	**5** (19) (5–5)	**5** (16) (5–15)	0.16	0.98
		**5** (12) (5–5)	**5** (12) (5–5)	**5** (7) (5–20)	NA	>0.99
**H1N1**	**Adjuvanted FLU-v**	**128** (48) (50–260)	**310** (47) (220–480)	**240** (39) (95–400)	**<0.0001**	**0.0163**
		**50** (47) (20–115)	**240** (46) (95–380)	**90** (40) (30–160)	**<0.0001**	0.16
		**25** (28) (5–45)	**125** (25) (30–170)	**50** (21) (20–155)	**0.0011**	**0.0025**
	**Adjuvanted** **Placebo**	**265** (20) (75–410)	**153** (18) (110–360)	**160** (15) (70–390)	0.29	0.90
		**73** (18) (20–200)	**65** (16) (20–125)	**73** (14) (10–285)	0.76	0.62
		**23** (12) (10–70)	**28** (12) (10–80)	**30** (7) (5–155)	0.83	0.56
**H3N2**	**Adjuvanted FLU-v**	**268** (48) (140–585)	**555** (47) (355–695)	**490** (39) (230–740)	**0.0001**	0.12
		**193** (48) (95–340)	**485** (45) (360–630)	**215** (40) (105–460)	**0.0002**	0.94
		**135** (29) (10–200)	**220** (26) (155–385)	**205** (22) (60–350)	**0.0064**	**0.0449**
	**Adjuvanted** **Placebo**	**510** (21) (205–800)	**420** (19) (235–575)	**460** (16) (230–865)	0.25	>0.99
		**235** (19) (70–530)	**310** (17) (175–435)	**310** (15) (95–480)	0.49	0.61
		**175** (12) (75–245)	**138** (12) (75–230)	**200** (7) (110–410)	0.42	0.78
**H5N1**	**Adjuvanted FLU-v**	**460** (45) (175–550)	**668** (42) (530–810)	**585** (37) (355–845)	**0.0001**	**0.0381**
		**305** (44) (160–495)	**650** (41) (450–750)	**325** (38) (115–570)	**0.0041**	0.69
		**245** (28) (110–335)	**305** (24) (250–455)	**260** (21) (175–465)	**0.0220**	0.18
	**Adjuvanted** **Placebo**	**493** (20) (250–890)	**450** (17) (340–700)	**470** (14) (170–1060)	0.73	0.80
		**490** (18) (120–800)	**433** (16) (275–580)	**360** (13) (105–650)	0.98	0.89
		**135** (11) (50–830)	**230** (11) (80–725)	**263** (6) (130–410)	0.76	0.56
**H7N9**	**Adjuvanted** **FLU-v**	**130** (44) (75–210)	**268** (40) (160–350)	**215** (36) (70–280)	**0.0026**	0.27
		**50** (43) (15–100)	**140** (39) (55–180)	**50** (35) (15–105)	0.49	0.88
		**20** (28) (10–45)	**60** (23) (20–115)	**23** (20) (5–80)	0.06	0.47
	**Adjuvanted** **Placebo**	**150** (18) (40–430)	**195** (16) (80–455)	**140** (13) (30–265)	0.75	0.79
		**90** (18) (10–135)	**83** (16) (10–260)	**50** (12) (5–100)	0.99	0.58
		**20** (11) (5–170)	**35** (11) (5–100)	**30** (6) (5–45)	0.71	>0.99
**B**	**Adjuvanted FLU-v**	**130** (47) (75–200)	**243** (46) (110–310)	**135** (39) (60–230)	**0.0085**	0.63
		**25** (48) (10–75)	**75** (45) (45–120)	**40** (39) (15–75)	0.0601	0.27
		**10** (28) (5–20)	**30** (25) (15–50)	**20** (21) (10–50)	**0.0046**	0.21
	**Adjuvanted** **Placebo**	**133** (20) (40–270)	**165** (18) (70–375)	**100** (15) (40–265)	0.96	0.79
		**45** (19) (5–80)	**45** (16) (15–110)	**25** (14) (5–45)	0.99	0.49
		**10** (12) (5–35)	**15** (12) (5–40)	**10** (7) (5–50)	0.64	0.72

**Table 3:** Median fold increase in number of IFN-γ-producing, granzyme-producing, and double positive cells (SFU/million cells) in the adjuvanted vaccine and placebo group from day 0 to days 42 and 180. Cells were stimulated with a mix of four synthetic peptides that composed the FLU-v vaccine or with whole inactivated influenza strains (A/California/7/2009 (H1N1), A/Shanghai/24/1990 (H3N2), A/Vietnam/1194/2004 (NIBRG-14) (H5N1), A/Anhui/1/2013 (NIBRG-268) (H7N9), and B/Brisbane/9/2014 (Yamagata lineage)). Fold increase on days 42 and 180 was defined as the ratio between SFU counts on day 42 or 180, and SFU counts on day 0. *n* = number of samples to be included in the statistical analysis. CI = 95% confidence interval. The Mann–Whitney U test (MW) was used to compare fold increases between groups, and test the null hypothesis of equal distributions in the vaccine group and placebo group on day 42 and on day 180. Since we cannot reasonably assume that the distributions have the same shape, and only differ with respect to location, the Mann–Whitney U test cannot be interpreted as a comparison of medians. Significant differences are indicated with *p*-values in bold.

Antigen	Median Fold Increase (n) (CI) Day 42			Median Fold Increase (n) (CI) Day 180		
	Adjuvanted FLU-v IFN-γ Granzyme B Double Positive	Adjuvanted Placebo IFN-γ Granzyme B Double Positive	*p*-Value (MW)	Adjuvanted FLU-v IFN-γ Granzyme B Double Positive	Adjuvanted Placebo IFN-γ Granzyme B Double Positive	*p*-Value (MW)
**FLU-v**	**16.3** (48) (9.0–25.0)	**1.0** (21) (1.0–1.0)	**<0.0001**	**9.5** (40) (4.0–19.0)	**1.0** (17) (1.0–2.0)	**<0.0001**
	**3.5** (46) (2.0–9.0)	**1.0** (19) (0.5–1.0)	**<0.0001**	**2.0** (41) (1.0–5.0)	**1.0** (16) (0.4–3.0)	**0.0461**
	**3.0** (25) (1.0–8.0)	**1.0** (12) (1.0–1.0)	**0.0012**	**1.0** (21) (1.0–4.0)	**1.0** (7) (1.0–4.0)	0.30
**H1N1**	**2.3** (47) (1.8–3.1)	**0.8** (18) (0.5–2.0)	**0.0083**	**1.9** (39) (1.4–3.0)	**0.8** (15) (0.4–3.3)	0.23
	**3.5** (46) (2.1–4.4)	**1.0** (16) (0.5–3.0)	**0.0075**	**1.2** (40) (0.8–2.6)	**0.9** (14) (0.1–5.5)	0.52
	**2.9** (25) (2.0–5.0)	**1.0** (12) (0.6–1.3)	**0.0219**	**1.8** (21) (1.0–6.6)	**0.5** (7) (0.1–7.8)	0.11
**H3N2**	**1.7** (47) (1.3–2.0)	**0.8** (19) (0.5–1.5)	**0.0178**	**1.3** ((39) 0.8–2.5)	**1.2** (16) (0.2–2.2)	0.43
	**1.8** (45) (1.1–2.6)	**1.2** (17) (0.5–3.3)	0.33	**0.9** (40) (0.7–1.6)	**2.0** (15) (0.4–3.6)	0.39
	**1.7** (26) (1.0–2.6)	**0.9** (12) (0.6–1.4)	**0.0136**	**1.3** (22) (0.8–4.0)	**1.5** (7) (0.2–4.0)	0.56
**H5N1**	**1.7** (42) (1.2–2.4)	**1.0** (17) (0.7–1.7)	**0.0441**	**1.5** (37) (1.1–1.8)	**1.2** (14) (0.4–3.0)	0.57
	**1.5** (41) (1.3–2.6)	**0.9** (16) (0.7–2.6)	0.28	**0.9** (38) (0.5–1.6)	**1.6** (13) (0.6–3.2)	0.31
	**1.4** (24) (1.1–2.6)	**0.9** (11) (0.6–2.1)	0.25	**1.2** (21) (0.9–2.5)	**2.2** (6) (0.5–12.7)	0.60
**H7N9**	**2.2** (40) (1.2–3.3)	**0.9** (16) (0.5–4.3)	0.25	**1.2** (35) (0.9–3.0)	**1.1** (13) (0.2–3.5)	0.42
	**1.7** (39) (0.6–4.0)	**0.8** (16) (0.2–11.0)	0.62	**1.0** (35) (0.3–3.0)	**1.1** (12) (0.1–4.0)	0.75
	**2.1** (23) (0.5–3.3)	**0.6** (11) (0.3–10.0)	0.59	**1.0** (20) (1.0–2.0)	**1.6** (6) (0.0–9.0)	0.68
**B**	**1.5** (46) (1.0–2.5)	**1.2** (18) (0.6–2.3)	0.51	**1.3** (39) (0.6–2.3)	**1.1** (15) (0.3–3.5)	0.97
	**2.0** (45) (1.0–3.3)	**0.9** (16) (0.3–5.0)	0.24	**0.8** (39) (0.4–1.6)	**1.0** (14) (0.1–6.1)	0.88
	**2.0** (25) (1.0–6.0)	**0.8** (12) (0.5–2.0)	**0.0227**	**1.2** (21) (1.0–5.0)	**1.5** (7) (0.1–5.0)	0.61

## Data Availability

Archives of the collected data are not publicly available.
